# Supplementary figures and images for: The NHGRI-EBI GWAS Catalog: standards for reusability, sustainability and diversity
Source: bioRxiv. 2024 Oct 23:2024.10.23.619767. Preprint. [Version 1] doi: 10.1101/2024.10.23.619767 (PMC11526975; doi:10.1101/2024.10.23.619767)

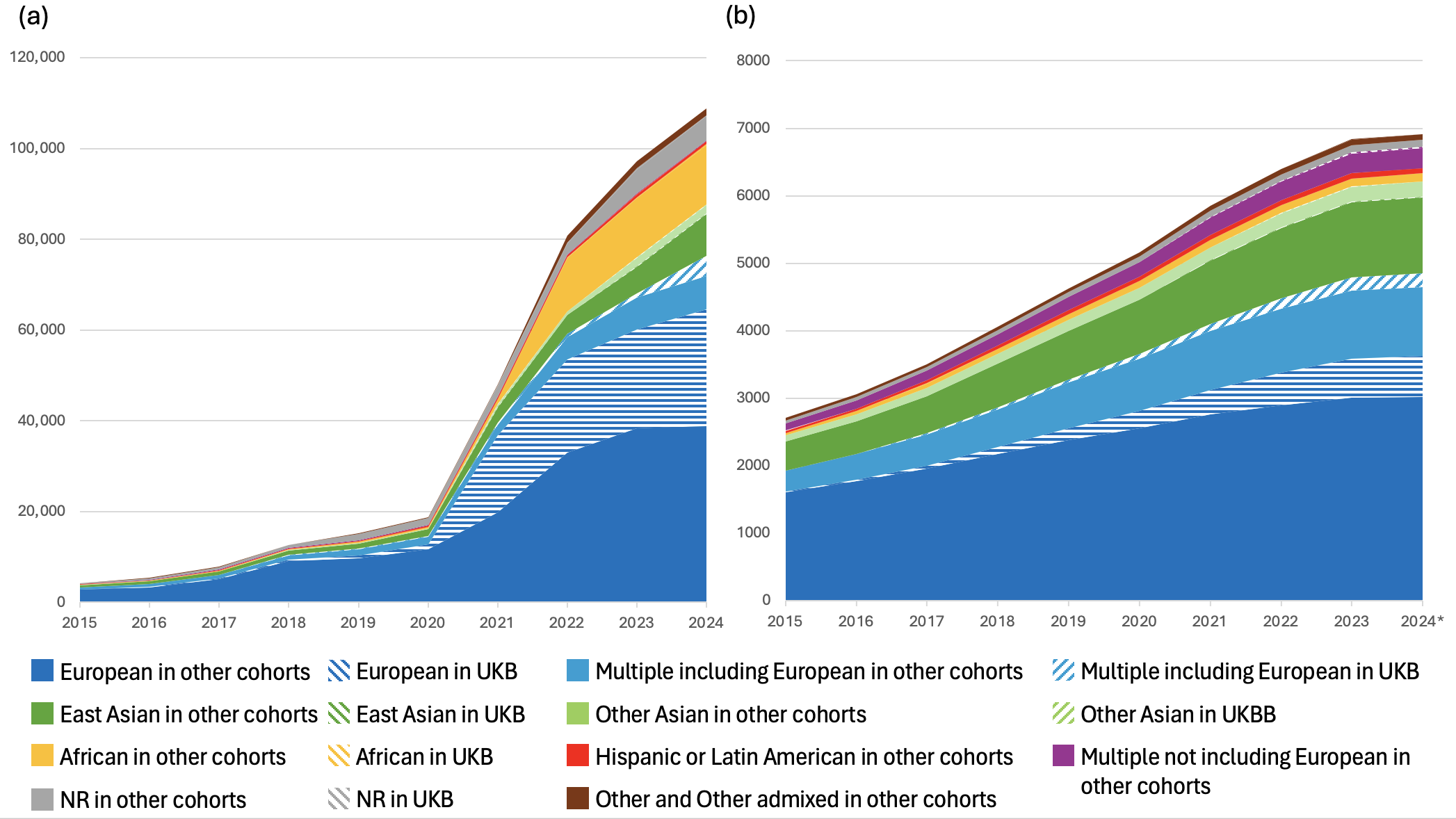

Supplement: Supplement 2 [file media-2.tif]
